# Comparative AAV-eGFP Transgene Expression Using Vector Serotypes 1–9, 7m8, and 8b in Human Pluripotent Stem Cells, RPEs, and Human and Rat Cortical Neurons

**DOI:** 10.1155/2019/7281912

**Published:** 2019-01-17

**Authors:** Thu T. Duong, James Lim, Vidyullatha Vasireddy, Tyler Papp, Hung Nguyen, Lanfranco Leo, Jieyan Pan, Shangzhen Zhou, H. Isaac Chen, Jean Bennett, Jason A. Mills

**Affiliations:** ^1^F.M. Kirby Center for Molecular Ophthalmology and Center for Advanced Retinal and Ocular Therapeutics (CAROT), Scheie Eye Institute, University of Pennsylvania Perelman School of Medicine, Pennsylvania, PA 19104, USA; ^2^Department of Neurosurgery, University of Pennsylvania, Philadelphia, Pennsylvania 19104, USA; ^3^Department of Computer and Information Science, University of Pennsylvania, PA 19104, USA; ^4^Corporal Michael J. Crescenz Veterans Affairs Medical Center, Philadelphia, Pennsylvania 19104, USA

## Abstract

Recombinant adeno-associated virus (rAAV), produced from a nonpathogenic parvovirus, has become an increasing popular vector for gene therapy applications in human clinical trials. However, transduction and transgene expression of rAAVs can differ across *in vitro* and ex vivo cellular transduction strategies. This study compared 11 rAAV serotypes, carrying one reporter transgene cassette containing a cytomegalovirus immediate-early enhancer (eCMV) and chicken beta actin (CBA) promoter driving the expression of an enhanced green-fluorescent protein (eGFP) gene, which was transduced into four different cell types: human iPSC, iPSC-derived RPE, iPSC-derived cortical, and dissociated embryonic day 18 rat cortical neurons. Each cell type was exposed to three multiplicity of infections (MOI: 1E4, 1E5, and 1E6 vg/cell). After 24, 48, 72, and 96 h posttransduction, GFP-expressing cells were examined and compared across dosage, time, and cell type. Retinal pigmented epithelium showed highest AAV-eGFP expression and iPSC cortical the lowest. At an MOI of 1E6 vg/cell, all serotypes show measurable levels of AAV-eGFP expression; moreover, AAV7m8 and AAV6 perform best across MOI and cell type. We conclude that serotype tropism is not only capsid dependent but also cell type plays a significant role in transgene expression dynamics.

## 1. Introduction

There is an excellent safety record with respect to use of recombinant adeno-associated virus (AAV) vectors in human clinical trials [1–3]. AAV-mediated gene therapy has been shown to rescue retinal and visual function in individuals diagnosed with inherited blinding disorders due to *RPE65* mutations [3–5]. rAAVs act by transferring the functional transgene cassette into the targeted cells or tissue where it can then be expressed. The specificity and efficiency of the AAV-mediated gene therapy significantly depend on cell type targeted, the number of vector particles delivered to the cell (and the number of particles that successfully reach the nucleus), immune response, and AAV capsid serotype utilized.

Since animal models are not always available for a given disease (or may have an irrelevant phenotype), recent trend for evaluating proof-of-concept of gene therapy in laboratory studies has focused on use of induced pluripotent stem cell- (iPSC-) based cell models from affected individuals [6–9]. Such models can also be used to study pathologic mechanisms associated with known gene mutations. An advantage of using iPSCs for translational models is that these cells can be differentiated along pathways leading to the three germ layers; endoderm (liver, lung, and pancreas), mesoderm (blood, endothelium, and mesenchymal cells), and ectoderm (brain, skin, and eye). For ophthalmologic evaluation, cells can be differentiated to the retinal cell lineage for the generation of retinal progenitors, retinal pigmented epithelium, retinal ganglion cells, horizontal, amacrine cells, and photoreceptors. These long-term cultures allow for developmental and pathophysiologic modeling. Due to the inaccessibility of the human brain, surrogate *in vitro-*derived patient cells are valuable for studying neurological disorders, which can affect numerous regions of the brain. The generation of individualized cell types from pluripotent stem cells offers a tremendous tool for developing rAAV therapeutics for ailing individuals; however, comparison of results in *in vitro* cell-based assays to *in vivo* or ex vivo models is necessary to ensure the relevance of gene augmentation strategies for ultimate human clinical trials.

The evolution of AAV capsid modifications has made available a new repertoire of vectors with different cell-type tropisms which are valuable for controlling transgene level, onset of expression, viral dosage, and organ- or cell-type specificity. In addition, the transduction efficiency *in vivo* versus *in vitro* is hard to predict without direct screening due to complex mechanisms in targeting the cellular receptors on different cell types and species. Although AAV serotype 2 (AAV2) is the most studied with respect to safety and efficacy and is the first AAV vector approved as a drug for the treatment of a retinal disease, other AAV serotypes have great potential and their utility needs to be fully elucidated [1, 2, 10, 11].

Here, our goal is to establish and optimize a methodological approach to identify which of a variety of AAV serotypes is best suited for applications *in vitro* and ex vivo by defining distributive parameters of AAV-eGFP transgene expression across AAV serotypes 1-9, 7m8, and 8b in numerous cellular models including in iPSC, iPSC-derived RPE, iPSC-derived cortical neurons, and dissociated embryonic day 18 (E18) rat cortical neurons to benefit proof-of-concept AAV translational research. Here, we conclude that AAV7m8 has superior performance for the particular cell types and duration evaluated; however, depending on dosage or time of onset additional serotypes like AAV6 and AAV8 have useful properties.

## 2. Methods

### 2.1. Human-Induced Pluripotent Stem Cells Culturing

Two iPSC cell lines (JBWT2 (PBWT2) and JBWT4 (BMC1)) derived from adult individuals without any known disease were evaluated in these studies and have been previously published showing a complete analysis of pluripotent stem characteristics [12, 13]. All human protocols were approved by the institutional review board (IRB) at the University of Pennsylvania, and each donor provided signed informed consent (IRB 808828). iPSC cultures were maintained in iPSC medium (Dulbecco's modified essential medium/Ham's F12 nutrient media; DMEM/F12 (50 : 50; Corning)) containing glutamine, penicillin/streptomycin (Invitrogen), 15% KnockOut serum replacement (KSR; Invitrogen), 1 × non-essential amino acids (NEAA; Invitrogen), 0.1 mM *β*-mercaptoethanol (2-ME; Invitrogen), and 5 ng/mL of basic fibroblast growth factor (bFGF; R&D Systems) on 0.1% gelatin-coated dishes with irradiated mouse embryonic fibroblast (iMEFs). For transduction experiments, cells were maintained on 1 : 100 Matrigel-coated 96-well dishes. Morphological characteristics and flow cytometry of pluripotency markers were evaluated prior to all experiments. Single cells were stained with stage-specific embryonic antigen 3 and 4 (SSEA3: BioLegend 1 : 100, SSEA4: BioLegend) for 30 minutes at room temperature. Following staining and three subsequent washes, cells were resuspended in FACS buffer (1XPBS (Corning) with 0.1% BSA (Sigma-Aldrich) and 0.1% sodium azide (Sigma-Aldrich)). All samples were analyzed on the Accuri C6 (BD Biosciences) software program.

### 2.2. Retinal Pigmented Epithelium (RPE) Cells

Retinal pigmented epithelium cells were generated from both iPS cell lines and fully characterized as previously published [14]. Cells were maintained in X-VIVO 10 medium (Lonza) plus 1 × pen/strep and 1 × glutamax on tissue culture dishes coated with 1 : 100 growth factor reduced (GFR) Matrigel (Corning). All passaging of RPE were performed using Accutase and replating media contained Thiazovivin (2 *μ*M; Stemgent), which was subsequently removed from X-VIVO media 24 h postsplitting. Monolayer cultures were characterized for RPE morphology and images were taken with Nikon D5600 SLR camera, and RPE were stained for cellular markers. RPE were cultured on 1 : 3 Matrigel-coated dishes, and cells were fixed in 4% paraformaldehyde (PFA; electron microscopy) for 15 minutes at room temperature. Cells were blocked for one hour (5% normal goat serum (CST), 2% BSA (Sigma-Aldrich), and 0.3% Triton X-100 in PBS) and stained with primary antibodies: zonula occludens-1 (Zo-1: Invitrogen 1 : 200) and microphthalmia-associated transcription factor (MITF: Invitrogen 1 : 200) in 5% normal goat serum, 2% BSA, and 0.1% Triton X-100 in PBS overnight. After washing, cells were stained with secondary antibodies (1 : 500, anti-rabbit 488 and anti-mouse 555, Invitrogen) in staining buffer for 2 hours at room temperature. The cells were counterstained with ProLong Gold with DAPI (ThermoFisher). Immunofluorescent images were captured with a Zeiss Axio Imager M2 microscope equipped with epifluorescence and AxioVision 4.6 software. For intracellular staining, cells were fixed using BD perm/fix (BD Biosciences) for 20 minutes at 4°C. Cells were washed and resuspended in saponin buffer (BioLegend). Primary and secondary antibodies for MITF (1 : 50, ThermoFisher) and goat anti-mouse 647 were diluted in saponin buffer, and cells were stained for 30 minutes at room temperature. Isotype controls (mouse, Invitrogen) were used at same protein concentration as primary antibodies and were used as negative controls. Following staining, cells were resuspended in FACS buffer and analyzed on Accuri C6 software program.

### 2.3. Human-Induced Pluripotent Stem Cell-Derived Cortical Neurons

For the iPSC-cortical neuron differentiations, on day 3, cells were dissociated using TrypLE (Invitrogen) and plated on GFR Matrigel in HES : MEF-conditioned (90 : 10) medium +20 ng/mL of bFGF and supplemented with ROCK inhibitor (10 *μ*M; Y27632, Tocris). Cells were maintained in this media until reaching 100% confluence (day 0). The differentiation was performed as previously published with some modifications [15, 16]. Briefly, on day 0, medium was changed to defined differentiation media (DDM) (DMEM/F12 (50 : 50), 1% N2 (Invitrogen), 2% B27 without vitamin A (Invitrogen), 1 × glutamax, 1 × pen/strep, 1X NEAA supplemented with XAV939 (XAV; 2 *μ*M, Stemgent), SB431542 (SB; 10 *μ*M, Tocris), and LDN (LDN; 100 nM, Stemgent). The medium was replenished daily until day 2. On day 2, cyclopamine (Cyclo; 1 *μ*M, Stemgent) was added to XAV, SB, and LDN. From day 2-10, the medium was replaced daily with DDM plus XAV, SB, LDN, and Cyclo. On days 10-16, DDM plus LDN only was used at daily feeding. Day 16-24, DDM was fed daily. From day 27, 25% of DDM was replaced with Neurobasal media (Invitrogen) every other day until day 33 when 100% Neurobasal was used to maintain long-term cortical cultures. On day 82, cortical cultures were characterized by immunofluorescence using Beta-Tubulin III (Tuj1: Sigma 1 : 1000), special AT-rich sequence-binding protein 2 (Satb2: Abcam 1 : 500) and Coup-TF-interacting protein 2 (CTIP2: Abcam 1 : 1000), and glial fibrillary acidic protein (GFAP: SCBT 1 : 300) as described above. Images were captured using a Nikon Eclipse Ti-2 inverted microscope, NIS Elements Advanced Research software and magnification of 10x.

### 2.4. Isolation of Rat Cortical Neurons

Rat cortical neuron experiments were performed using isolated cells from embryonic day 18 Sprague-Dawley rat fetuses. All animal experiments were performed in agreement with the National Institutes of Health guidelines and approved by the University of Pennsylvania Institutional Animal Care and Use Committee (IACUC). Rat fetuses were removed from euthanized timed-pregnant dames via caesarean section. Fetuses were then decapitated, and their brains isolated. Cortical neurons were isolated from 4-6 cortical hemispheres, which were dissociated with 0.25% Trypsin/EDTA (Invitrogen) containing DNase I (Roche) at 37°C. Cells were washed three times using HBSS and suspended in neuronal media: Neurobasal media plus 2% B27, 1 × glutamine, and 1 × pen/strep. Analysis of rat cortical neurons were performed to confirm identity using immunofluorescence of Tju1 (Sigma, 1 : 1000), Satb2 (Abcam, 1 : 500), and CTIP2 (Abcam, 1 : 1000) cortical cell markers and GFAP (SCBT: 1 : 300) as described above. Images were captured using a Nikon Eclipse Ti-S inverted microscope, NIS Elements Advanced Research software and magnification of 10x.

### 2.5. Generation of a Panel of Recombinant Adeno-Associated Virus (AAV) Serotypes Carrying Enhanced GFP cDNA Driven by CMV.C*β*A Promoter

A panel of 11 recombinant AAV serotypes were generated by the Center for Advanced Retinal and Ocular Therapeutic (CAROT) research vector core. Triple transfection of HEK293T cells with pAAV.CMV.C*β*A.eGFP and helper plasmids was performed as previously described [9]. The virus was purified by CsCl2 gradient centrifugation, and viral particles were titered by silver staining as vector genome copies/mL [17]. The virus was aliquoted in 0.001% Pluronic F-68 (formulation buffer) in 1 × PBS as single use aliquots and stored at -80°C prior to experimentation.

### 2.6. AAV Transduction Testing

For transduction experiments, 96-well plates were coated with 1 : 100 GFR Matrigel overnight. Cells were enzymatically detached to create a single-cell suspension prior to plating at a density of approximately 2E4 for iPSC, 8E4 iPSC-RPE, 1E5 iPSC-cortical neuron, and 2E4 rat cortical neuron per well. After 24 to 48 h, cells from a minimum of three wells per dish were disassociated and cell counts were performed. Transduction of rAAV-eGFP serotypes, AAV1, AAV2, AAV3, AAV4, AAV5, AAV6, AAV7, AAV7m8, AAV8, AAV8b, and AAV9, was executed at concentrations of 0, 10,000 (1E4), 100,000 (1E5), and 1,000,000 (1E6) vector genomes per cell (vg/cell) in triplicate. The amount of virus needed to achieve a multiplicity of infection (MOI: 1E4, 1E5, or 1E6 vg/cell) was based on the cell number per well for each AAV serotype. Viral stocks were diluted in 0.001% Pluronic F-68 (formulation buffer) in 1 × PBS when necessary. Representative example of dilution strategy is provided in Supplemental Table 1. For each experiment, a new fresh vial of viral stock was thawed and immediately used for transduction experiments. The day of transduction was considered 0 h. Viral particles were incubated for 24 h at 37°C and 5% CO_2_ for RPE and cortical cells and 5% CO_2_ and 5% O_2_ for iPSC and then replaced with corresponding media for each cell type. The AAV-eGFP expression was measured at four time points (24, 48, 72, and 96 h) postinfection using the Typhoon scanner (GE Healthcare) set for 520, BP40 for the emission filter, and 488 for the excitation filter, PTM 570, focal plane +3 mm. The densities of AAV-eGFP expressing cells were evaluated by ImageJ image processing software (FIJI, protein analyzer). Data represent mean relative fluorescence unit (RFU) in AAV-eGFP fluorescence between three technical replicates per experiment. Data from each group were normalized to the mean of nontransduced control wells. Experiments were performed at least three times. AAV-eGFP expression levels for 1E6 vg/cell was plotted using the scatter plot, 3D bubble plot in excel, to show relative differences in fluorescence between cell types and serotype on a per cell basis.

### 2.7. Bioinformatics and Statistics

The data of relative fluorescent per cell at three different MOIs were visualized by heat map (i.e., two-dimensional representations in colors). The heat map of each cell type was generated using Seaborn, a visualization library based on Python matplotlib. Fluorescent per cell measurements were parsed from excel datasheets into floating-point vectors with each vector corresponded to all AAV serotypes in each MOI over multiple postinfection measurement times. The colors were based on the relative fluorescent per cell level and ranged from yellow (low) to brown (high) fluorescent intensity. Analysis of variance (ANOVA) (Excel's Analysis ToolPak) for repeated measures was performed to determine the relationship of cell lines and AAV serotypes on the gene expression effect. To determine the effect of each AAV serotype for each cell line, ANOVA with one factor (i.e., AAV serotype) was used, followed by the post hoc pairwise comparisons (XLSTAT add-ins) with correction for multiple comparisons using Tukey method. For each AAV serotype, the gene expression post-infection across time (48, 72 and 96 h post-infection) was evaluated using repeated measures ANOVA followed by pairwise comparison with correction for multiple comparisons using Tukey method. The differences were considered statistically significant when *p*value < 0.05^∗^, <0.01^∗∗^, and <0.001^∗∗∗^ after correction for multiple comparisons using Tukey method.

## 3. Results and Discussion

### 3.1. AAV Tropism in Pluripotent Stem Cells, Derivatives, and Ex Vivo-Isolated Neurons

**Figure 1:**
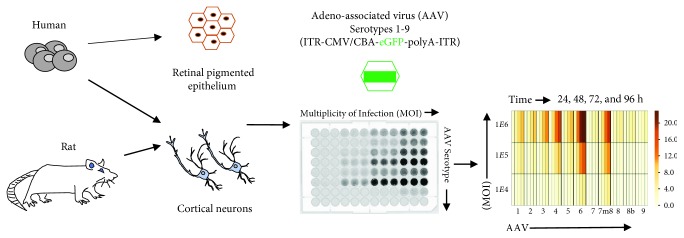
Schematic of rAAV tropism testing for *in vitro* and ex vivo-isolated cells. Human *in vitro* cellular models (including induced pluripotent stem cells (iPSC), iPSC-derived retinal pigment epithelium (iPSC-RPE), and iPSC-derived human cortical neurons) and *ex vivo*-isolated rat cortical neurons were generated and cultured. Before transduction testing, cells were collected and seeded at equal numbers in each well of two Matrigel-coated 96-well plates. Eleven AAV serotypes at different multiplicity of infection (MOI) were introduced to the cells in triplicate. GFP expression was measured by scanning the plate in Typhoon scanner followed by an analysis with protein array analyzer on ImageJ. The AAV-eGFP transgene expression was normalized with background and used as data to compare the critical effect of cell types and AAV serotypes on transgene expression.

Pluripotent stem cells can be used to generate numerous cell types which are candidates for rAAV vector studies and disease modeling. Induced pluripotent stem cells express > 20,000 mRNA transcripts [18], many of which are only expressed in the pluripotent state or specific adult cell type. These cells provide a resource for studying gene transfer effects on health and disease. Such studies have been the impetus for initiation of clinical trials for choroideremia [9, 14, 19]. However, AAV tropism in iPSCs and cells derived from them has not been thoroughly explored. Furthermore, the effects of AAV delivery to iPSC-derived cells with ex vivo-isolated cell types have yet to be compared across equivalent cell types. Here, we examined the level of AAV-eGFP expression following the transduction of eleven recombinant AAV (rAAV) vectors on iPSCs, iPSC-derived RPE, iPSC-derived cortical neurons, and *ex vivo*-isolated rat cortical neurons. The rAAV vectors were generated by packaging a transgene cassette encoding enhanced GFP with a bgh poly(A) sequence driven by the CMV.CßA promoter into the 11 different AAV serotypes. The AAV-eGFP transgene expression was determined at four time points and three MOIs and expressed as relative AAV-eGFP intensity per cell (Figure 1).

### 3.2. Pattern and Fluorescent Intensity of AAV-eGFP Differed between iPSCs and iPSC-Derived RPEs for rAAV Transductions

**Figure 2:**
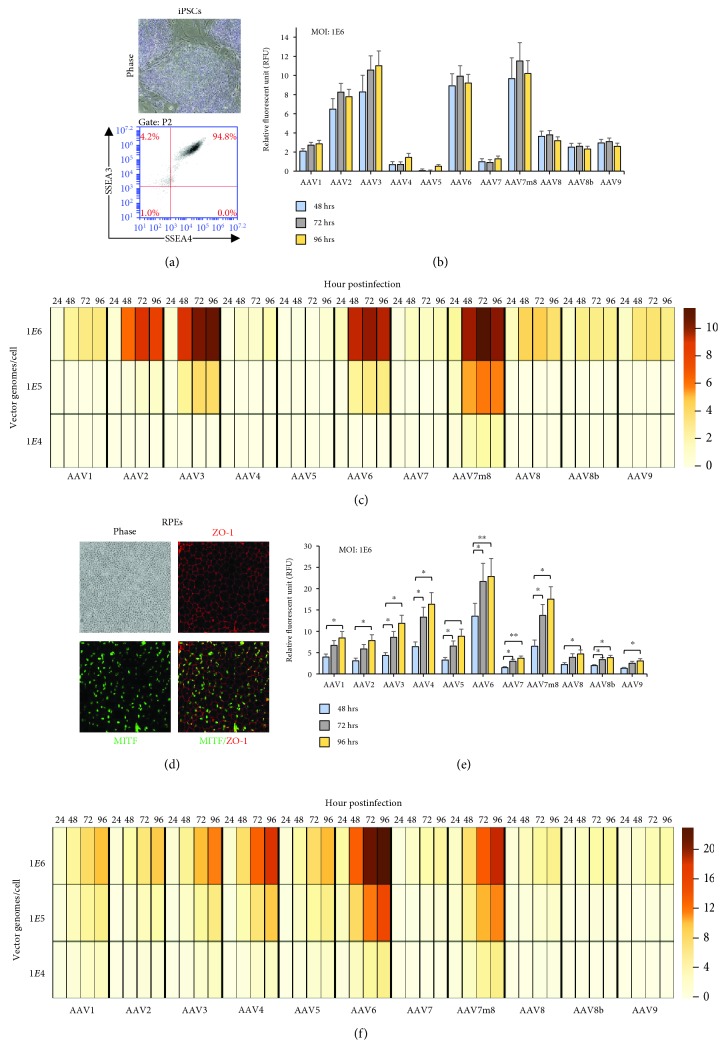
iPSC and iPSC-RPE tropism of 11 rAAV serotypes. (a) Representative phase image showing morphology of IPSC cultures and flow cytometry analysis of pluripotency surface marker expression (SSEA3 and SSEA4) of iPS cells. (b) The onset of AAV-eGFP expression in iPSCs at a dosage of 1E6 vg/cell for all rAAV at 48, 72, and 96 h posttransduction. There is no significant difference between any two time points (48, 72, and 96 h) after correction for multiple comparisons using Tukey method. (c) Heat map showing relative AAV-GFP expression per cell across all rAAV, dosages, and time in iPSCs. The scale bar shows the intensity of AAV-eGFP expression presented as an arbitrary relative fluorescence unit (A.U.) per cell. (d) Retinal pigmented epithelium (RPE) showing “cobblestone” appearance in phase (10x magnification), and immunofluorescent labeling with expression of ZO-1 (red) and MITF (green), and merged images showing uniform RPE monolayer. Images were captured at 10x magnification. (e) AAV-eGFP expression in RPEs at a dosage of 1E6 vg/cell for all rAAV at 48, 72, and 96 h postinfection. Analysis of variance for repeated measures with post hoc pairwise comparisons between time points were performed with statistical significance indicated with *p*value < 0.05^∗^, <0.01^∗∗^, and <0.001^∗∗∗^ after correction for multiple comparisons using Tukey method. (f) Heat map showing relative AAV-eGFP expression per cell across all rAAV, dosages, and time in RPE cells.

Analysis of AAV tropism was performed on iPSCs derived from two individuals, and cell lines were cultured in undifferentiated cell culture conditions to maintain a pluripotent state. Prior to transduction, to ensure cultures were a uniform population of pluripotent stem cells (PSC), cultures were monitored for PSC morphological characteristics (phase) and pluripotent surface markers expression of SSEA3 and SSEA4, by flow cytometry (Figure 2(a)). Aliquots of iPSC cultures were transduced with one of 11 different serotypes and MOIs of 1E4, 1E5, and 1E6 vg/cell. The AAV-eGFP expression postinfection was recorded using live cell imaging using a Typhoon fluorescent plate reader at 24, 48, 72, and 96 h. The expression of GFP was observed to be dose and time dependent, and only at the highest dose (MOI of 1E6) were all 11 rAAV expressed to measurable levels (Figures 2(b) and 2(c); *p* < 0.0001; Figure 2(e)). AAV-eGFP expression was first noticeable at approximately 48 h postinfection, and the level of expression was maintained throughout the 96 h of experimentation. It is important to note that the iPSCs after 96 h are 100% confluent resulting in spontaneous differentiation; therefore, the evaluation at later time points of the pluripotent state is no longer possible. There were significant differences across rAAV vectors used, and the expression of AAV-eGFP from highest to lowest was as follows at MOI: 1E6 vg/cells: AAV3, AAV7m8, AAV6, AAV2, AAV8, AAV1, AAV9, AAV8b, AAV4, AAV7, and AAV5 (Figure 2; Supplemental Table 2). AAV7m8 was the only serotype to yield expression across all vector dosages (1E4, 1E5, and 1E6 vg/cell). AAV3 and AAV6 were the only other rAAVs to yield expression at more than one dosage (1E5 and 1E6 vg/cell). Identifying the appropriate AAV vector for gene delivery in pluripotent stem cells (PSCs) is important, and expanding our repertoire of tools is essential for studies probing the biology in health and disease. Previous work has investigated the use of AAV1, AAV2, AAV6, and AAV9 for transduction of iPSC and demonstrated that AAV2 and AAV6 have high tropism for pluripotent stem cells [20]. Our work supports these findings and shows that AAV3 and AAV7m8 yield substantially increased GFP expression. Moreover, the characteristics of AAV transduction with respect to pluripotent stem cells differentiated to ocular and brain cell types will be pivotal for a number of translational studies as these areas are prime targets for gene augmentation strategies prerequisite to gene therapy human clinical trials.

RPE cells have been subject to a number of clinical interventions for the treatment of vision disorders such as retinitis pigmentosa (RP), Leber's congenital amarosis (LCA), advanced neovascular age-related macular degeneration, and choroidal neovascularization [10, 21–23]. The differentiation of iPSCs to retinal pigmented epithelium (RPE) cells provides for structurally and functionally valuable tools for genetic and translational studies [19, 24–26]. The predominant rAAVs for vector delivery to RPE have been AAV2 and AAV5, which are shown to transduce RPE with high efficiency [19]. However, AAV7m8 has proved to be an excellent candidate for both *in vitro* and *in vivo* studies based on its tropism for iPSC-RPE and nonhuman primate (NHP) retina using subretinal injections [14, 27]. Here, we expand the list of rAAV serotypes available for *in vitro* transduction of human iPSC-RPEs. Analysis of iPSC-RPEs prior to transduction experiments demonstrated the characteristic “cobblestone” appearance under phase contrast microscopy and colabeling with MITF and Zo-1 using immunohistochemistry showed distinct nuclear and cytoplasmic staining of RPE markers in >95% of cultured cells, respectively, and confirmed by intracellular flow cytometry analysis of MITF (Figure 2(d), Supplementary Figure 1(a)). Similar to iPSCs, there were time, dosage, and vector effects observed in iPSC-RPEs after administration of 1E6 vg/cell (^∗^ *p* < 0.05). We identified 1E6 to be the only dosage with detectable AAV-eGFP expression among all rAAV serotypes (Figure 2(e)). AAV-eGFP fluorescence did not appear until 48 h, similar to iPSCs; however, there was a time-dependent expression effect with maximal fluorescence intensity per cell being reached at 96 h postinfection. This time, delay may relate to the time needed to convert the single stranded DNA in AAV to a double-stranded transcriptionally competent form [28, 29]. Similar time delays have been noted after subretinal delivery *in vivo* [30].

RPE cells were able to be maintained on culture dishes for months postinfection and eGFP expression was also maintained; moreover, the level of transgene expression remained consistent with expression measured at 96 h posttransduction for weeks (Supplementary Figure 2). In the majority of the rAAV serotypes administered, there was noticeable AAV-eGFP expression at the lowest MOI (1E4 vg/cell), but expression was increased by more than ~3-fold (1E5 vg/cell) and ~7-fold (1E6 vg/cell) at the higher MOIs across most rAAVs (Figure 2(f)). The increases in intensity or AAV-eGFP expression were not directly associated with the vector copy number increase (10 and 100x, respectively), which could reflect a threshold for AAV binding to receptors, endocytosis mechanisms, and postentry processing of the capsids presented to RPE cells. A pattern of AAV-eGFP expression was observed in RPEs from highest to lowest were as follows at MOI: 1E6 vg/cells: AAV6, AAV7m8, AAV4, AAV3, AAV5, AAV1, AAV2, AAV8, AAV8B, AAV7, and AAV9 (Figure 2, Supplemental Table 2). The greatest fluorescent intensity of AAV6-eGFP was unexpected as this vector likely arose from recombination between AAV1 and AAV2 [31]. However, it has been used successfully in a previous study to deliver a transgene *in vivo* to RPE cells [32]. As previously reported, *in vivo* comparative studies in murine, rat, canine, and NHP models have shown AAV7m8, AAV2, and AAV4 to have superior transduction after subretinal AAV administration [27, 32–34]. Our studies demonstrate a correlation of *in vitro* findings in iPSC-RPEs with *in vivo* transduction of RPEs and thus show the utility of iPSC-RPEs as a surrogate for gene delivery effects *in vivo*.

### 3.3. AAV Tropism in Cortical Neurons Derived from iPSC and Ex Vivo Rat Cortical Neurons

**Figure 3:**
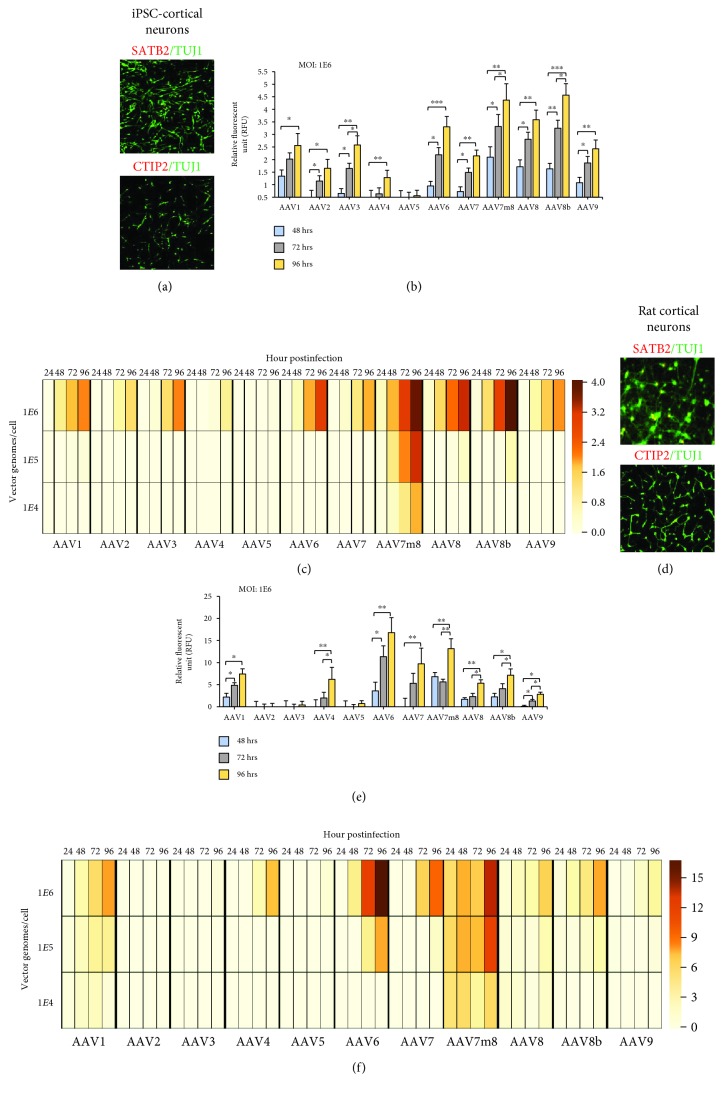
rAAV tropism of in vitro-derived cortical neurons compared to ex vivo-isolated rat cortical neurons. (a) iPSC-derived cortical neurons show dorsal forebrain identity at 82 days in vitro (DIV). The expression of pyramidal neuron markers, Tuj1 (green)/Satb2 (red) or Tuj1 (green)/CTIP2 (red), is shown. Images were captured at 10x magnification. (b) The onset of GFP expression in iPSC-derived cortical neurons (82-86 DIV) at a dosage of 1E6 vg/cell for all rAAV at 48, 72, and 96 h postinfection. Analysis of variance with post hoc pairwise comparisons between time points were performed with statistical significance indicated with *p*value < 0.05^∗^, <0.01^∗∗^, and <0.001^∗∗∗^ after correction for multiple comparisons using Tukey method. (c) The relative AAV-eGFP per cells was observed to be lowest in iPSC-cortical neurons compared to all cells analyzed. The heat map showing relative AAV-GFP expression per cell across all rAAV, dosages, and time in iPSC-derived cortical neurons. The scale bar shows the intensity of AAV-eGFP expression presented as an arbitrary relative fluorescence unit (R.F.U.) per cell. (d) *Ex vivo* cortical rat neurons were isolated at embryonic day 18. Similar to iPSC-cortical neurons expression, rat cortical neurons express Tuj1 (green)/Satb2 (red) or Tuj1 (green)/CTIP2 (red). Images were captured at 10x magnification. (e) AAV-eGFP expression in rat cortical neurons at a dosage of 1E6 vg/cell for all rAAV at 48, 72, and 96 h. Analysis of variance with post hoc pairwise comparisons between time points were performed with statistical significance indicated with *p*value < 0.05^∗^, <0.01^∗∗^, and <0.001^∗∗∗^ after correction for multiple comparisons using Tukey method. (f) Rat cortical neurons show the highest AAV-GFP expression among cell types, and heat map shows the relative AAV-GFP expression per cell across all rAAV, dosages, and time in rat cortical cells.

Cortical neurons were generated from the same iPS cells as described above, and the phenotype of these neurons (day 82+) was determined by immunocytochemical staining with markers of supragranular (layers II/III; Satb2) and infragranular (layer V; CTIP2) neurons prior to AAV transduction experiments. Within these cultures, two different cell types were predominant, either postmitotic pyramidal neurons (Tuj1+, 84.7%±12) or astrocytes (GFAP+, 4%±0.34) as identified by immunocytochemical analysis (Figure 3(a), Supplementary Figure 2(b)). For a comparison of iPSC-derived cortical neurons to *ex vivo*-isolated neurons, we isolated cortical neurons from rats at embryonic day 18 and stained with Satb2/CTIP2/TUJ1/GFAP (Figure 3(d), Supplementary Figure 2(c)). Similar to differentiated human cortical neurons, the majority of rat cortical cultures were predominantly cortical neurons expressing Tuj1 (88.2%±10) and astrocytes expressing GFAP (3%±1).

**Figure 4:**
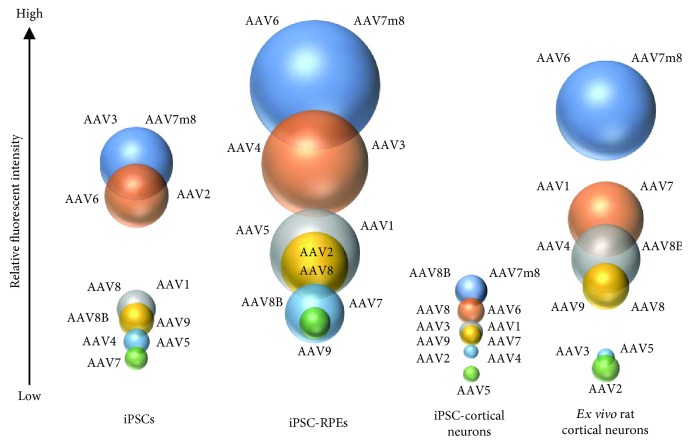
Overview of AAV-eGFP transgene expression across four cell types. The illustration shows cell type and serotype comparison between induced pluripotent stem cells (iPSC), iPSC-derived retinal pigmented epithelium (RPE), iPSC-derived cortical neurons, and *ex vivo*-isolated rat cortical neurons. The size of the bubble is proportional to the level of GFP expression measure within and across cell type.

The fluorescence intensity of eGFP for all rAAV serotypes was significantly lower (~2-4-fold, ^∗∗^ *p* < 0.01) for iPSC-cortical neurons compared to rat cortical cells. The best performing rAAV in iPSC neurons across time and dosage was AAV7m8 (Figure 3(b)). The highest AAV-eGFP expression was observed with AAV8B-eGFP, which was only significantly expressed at the highest MOI (1E6 vg/cell) and onset of expression was later at 96 h. We observed iPSC-cortical neuron AAV-eGFP expression from highest to lowest as follows at MOI: 1E6 vg/cells: AAV8b, AAV7m8, AAV8, AAV6, AAV3, AAV1, AAV9, AAV7, AAV2, AAV4, and AAV5 (Figure 3, Supplemental Table 2). This expression was time dependent with expression increasing across all times observed with maximal expression measured at 96 h (^∗^ *p* < 0.05). The AAV-eGFP expression was quite different between iPSC neurons and rat cortical neurons (Figure 4). The AAV-eGFP expression in rat cortical neurons from highest to lowest was as follows at MOI: 1E6 vg/cells: AAV6, AAV7m8, AAV7, AAV1, AAV8b, AAV4, AAV8, AAV9, AAV5, AAV3, and AAV2 (Figure 3, Supplemental Table 2). Previous work shows stable transgene expression when ex vivo hippocampal and cortical cultures were transduced with a panel of AAV serotype [35]. Similar to our studies, AAV6 and AAV1 are shown to be neurotrophic serotypes with high transduction efficiencies and transgene expression. The AAV7m8 serotype was found to have the highest AAV-eGFP expression across cortical neurons investigated.

### 3.4. rAAV Tropism Varies across Cell Types Resulting in Substantial Expression Differences

Although initial AAV tropism studies focused on AAV2, other serotypes have recently been shown to perform better *in vitro* and *in vivo* postadministration [11, 27, 36, 37]. We show that rAAV packaged using 11 different serotypes and one transgene cassette have substantial differences between cell type with respect to level of eGFP expression and time of onset. In particular, AAV7m8 is the leading candidate for transduction across all cell types and MOIs examined in these studies. The expression difference using AAV7m8 can range from 4 to 17-fold within the same vector genome copy number using the same transgene vector. AAV6 had significant expression across all cell types thereby highlighting its potential for advancing mechanistic and translational studies; additionally, AAV8 serotypes have efficacy in neuronal subtypes. To compare the level of AAV-eGFP expression across cell types, we evaluated AAV-eGPF expression on a per cell basis. The cell type most strongly transduced was the retinal pigmented epithelium cell compared to the other cell types (Figure 4). *Ex vivo* rat cortical neurons were observed to have high level of GFP expression compared to *in vitro* iPSC-derived cortical neurons. However, due to species differences, it is difficult to determine if AAV-eGFP expression levels in neurons are due to promoter activity or as result of serotype difference. iPSCs were shown to have low to intermediate level of GFP expression, which was serotype dependent. The performance of individual AAV serotypes differs significantly presumably due to capsid proteins; however, the individual cell tropism has been suggested to be receptor mediated and endocytosis or intracellular pathway related [38–40]. This data indicates that cell type-specific difference rather than AAV capsid affects the AAV tropism.

Important to note, having the highest level of AAV-eGFP expression will not always provide optimal results as the biology associated with transgene element needs to be considered. The endogenous level and transgene function, transcription factor, structural protein, chaperone, and enzymes, all need to be evaluated during gene augmentation testing. Evaluation of gene expression kinetic profiles also provides insight in the appropriate timing for the evaluation of maximal and sustained AAV-transgene expression which can be used for preclinical and clinical viral preparation validation. Therefore, having a group of rAAVs with variable gene expression kinetic, protein expression profiles, and cell tropisms is essential for selecting the appropriate levels of transgene expression for a given application.

## Data Availability

The data used to support the findings of this study are available from the corresponding author upon request.

## Supplementary Materials

Supplementary Materials Supplemental Table 1: representative AAV dosage, titer, and MOI calculation for iPSC, RPE, and human and rat cortical cells. Supplemental Table 2: relative fluorescence intensity (R.F.U.) per cell at 96 h at MOI (1E6 vg/cell) Supplementary Figure 1: stable GFP transgene expression in RPE. (a) The AAVeGFP expression in RPE at d4 (96 h) and d18 for a dosage of 1E6 vg/cell shows similar expression in longer term cultures. There is no statistically significant difference between day 4 and day 18 in any of the AAV. Supplementary Figure 2: RPE and cortical neuron characterization. (a) Flow cytometric analysis of intracellular MITF protein expression. Greater than 95% of RPE expresses MITF. Plot showing MITF positive cells (blue) relative to mouse isotype control antibody (red). (b, c) Immunophenotyping of human and rat cortical neurons and astrocytes showing neuron-specific class III beta-tubulin (TUJ1; green) and glial fibrillary acidic protein (GFAP; red), respectively. Images were captured at 20x magnification (human iPSC cortical) and 10x magnification (rat cortical).
