# Supplementary material for: Comparative AAV-eGFP Transgene Expression Using Vector Serotypes 1–9, 7m8, and 8b in Human Pluripotent Stem Cells, RPEs, and Human and Rat Cortical Neurons
Source: Stem Cells Int. 2019 Jan 17;2019:7281912. doi: 10.1155/2019/7281912 (PMC6360060; doi:10.1155/2019/7281912)

Supplemental Table 1. Representative AAV dosage, titer and MOI calculation for iPSC, RPE, human and rat cortical cells.

Supplemental Table 2. Relative fluorescence intensity (R.F.U) per cell at 96h at MOI (1E6 vg/cell)

Supplementary Figure 1. Stable GFP transgene expression in RPE. (A) The AAV-eGFP expression in RPE at d4 (96h) and d18 for a dosage of 1E6 vg/cell show similar expression in longer term cultures. There is no statistically significance difference between day 4 and day 18 in any of the AAV.

Supplementary Figure 2. RPE and Cortical Neuron Characterization. (**A**) Flow cytometric analysis of intracellular MITF protein expression. Greater then 95% of RPE express MITF. Plot showing MITF positive cells (blue) relative to mouse isotype control antibody (red). (**B-C**) Immunophenotyping of human and rat cortical neurons and astrocytes showing neuron-specific class III beta-tubulin (TUJ1; green) and glial fibrillary acidic protein (GFAP; red), respectively. Images were capture at 20x magnification (human iPSC-cortical) and 10x magnification (rat cortical).

Supplemental Table 1: Representative AAV dosage, titer and MOI calculation for iPSC, RPE, human and rat cortical cells.

			iPSC			IPSC-RPE			Ex Vivo Rat Corticals			Human iPSC-derived Corticals		
			MOI (vector genomes/cell)			MOI (vector genomes/cell)			MOI (vector genomes/cell)			MOI (vector genomes/cell)		
**Serotypes**	**Lot Number**	**Titer**	**1E+04**	**1E+05**	**1E+06**	**1E+04**	**1E+05**	**1E+06**	**1E+04**	**1E+05**	**1E+06**	**1E+04**	**1E+05**	**1E+06**
AAV1-CMV/CBA-eGFP	CT 290	8.95E+12	2.3	2.3	2.3	8.8	8.8	8.8	22.3	22.3	22.3	11.2	11.2	11.2
AAV2-CMV/CBA-eGFP	CT 176	3.28E+12	6.4	6.4	6.4	24.0	24.0	24.0	61.0	61.0	61.0	30.5	30.5	30.5
AAV3-CMV-CB-eGFP	SAB-589	1.59E+13	1.3	1.3	1.3	5.0	5.0	5.0	12.6	12.6	12.6	6.3	6.3	6.3
AAV4-CMV/CBA-eGFP	CT 318	9.58E+12	2.2	2.2	2.2	8.2	8.2	8.2	20.9	20.9	20.9	10.4	10.4	10.4
AAV5-CMV/CBA-eGFP	CT 73	1.18E+13	1.8	1.8	1.8	6.7	6.7	6.7	16.9	16.9	16.9	8.5	8.5	8.5
AAV6-CMV/CBA-eGFP	CT 291	9.51E+12	2.2	2.2	2.2	8.3	8.3	8.3	21.0	21.0	21.0	10.5	10.5	10.5
AAV7-CMV/CBA-eGFP	CT 319	1.6E+13	1.3	1.3	1.3	4.9	4.9	4.9	12.5	12.5	12.5	6.3	6.3	6.3
AAV7m8-CMV/CBA-eGFP	SAB-608	6.51E+12	3.2	3.2	3.2	12.1	12.1	12.1	30.7	30.7	30.7	15.4	15.4	15.4
AAV8-CMV/CBA-eGFP	CT 118	1.11E+13	1.9	1.9	1.9	7.1	7.1	7.1	18.0	18.0	18.0	9.0	9.0	9.0
AAV8b-CMV/CBA-eGFP	CT 320	1.16E+13	1.8	1.8	1.8	6.8	6.8	6.8	17.2	17.2	17.2	8.6	8.6	8.6
AAV9-CMV/CBA-eGFP	CT 292	7.88E+12	2.7	2.7	2.7	10.0	10.0	10.0	25.4	25.4	25.4	12.7	12.7	12.7
	**Dilution ratio**		1:100	1:10	Stock	1:100	1:10	Stock	1:1000	1:100	1:10	1:100	1:10	Stock

**Cell Type**	**Cell Number**
IPSCs	21,000
IPSC-RPEs	78,750
Ex Vivo Rat Corticals	20,000
Human iPSC-Corticals	100,000

Supplemental Table 2. Relative fluorescence intensity (R.F.U) per cell at 96h at MOI (1E6 vg/cell)

**iPSC**			**iPSC-RPE**			**Human iPSC-Cortical Neurons**			**Ex Vivo Rat Cortical Neurons**		
**AAV type**	Mean§ R.F.U	SEM	**AAV type**	Mean§ R.F.U	SEM	**AAV type**	Mean§ R.F.U	SEM	**AAV type**	Mean§ R.F.U	SEM
AAV3	11.01[a]	± 1.55	AAV6	22.86[a]	± 4.23	AAV8b	4.06[a]	± 0.46	AAV6	16.79[a]	± 3.39
AAV7m8	10.19[a]	± 1.35	AAV7m8	17.54[ab]	± 2.91	AAV7m8	3.87[ab]	± 0.65	AAV7m8	13.15[ab]	± 2.23
AAV6	9.20[a]	± 0.91	AAV4	16.33[abc]	± 2.76	AAV8	3.09[abc]	± 0.38	AAV7	9.72[abc]	± 3.55
AAV2	7.77[a]	± 0.78	AAV3	11.86[bcd]	± 1.90	AAV6	2.81[abcd]	± 0.41	AAV1	7.42[bcd]	± 1.18
AAV8	3.18[b]	± 0.40	AAV5	8.82[bcd]	± 1.67	AAV3	2.08[bcde]	± 0.37	AAV8b	7.14[bcd]	± 1.42
AAV1	2.85[b]	± 0.36	AAV1	8.43[bcd]	± 1.51	AAV1	2.06[bcde]	± 0.48	AAV4	6.21[bcd]	± 2.71
AAV9	2.59[b]	± 0.34	AAV2	7.83[cd]	± 1.37	AAV9	1.93[cde]	± 0.35	AAV8	5.33[bcd]	± 0.77
AAV8b	2.30[b]	± 0.30	AAV8	4.68[d]	± 0.95	AAV7	1.65[cdef]	± 0.23	AAV9	2.82[cd]	± 0.48
AAV4	1.43[b]	± 0.42	AAV8b	3.82[d]	± 0.51	AAV2	1.15[def]	± 0.35	AAV5	0.73[cd]	± 0.64
AAV7	1.28[b]	± 0.30	AAV7	3.66[d]	± 0.53	AAV4	0.78[ef]	± 0.29	AAV3	0.40[d]	± 0.84
AAV5	0.52[b]	± 0.18	AAV9	3.04[d]	± 0.55	AAV5	0.06[f]	± 0.22	AAV2	0.00[d]	± 0.75
Pr > F(Model)	< 0.0001£		Pr > F(Model)	< 0.0001£		Pr > F(Model)	< 0.0001£		Pr > F(Model)	< 0.0001£	

*SEM: Standard error of mean

£For comparisons across 11 AAV groups to determine whether there are any statistically significant difference among them using one-way analysis of variance.

§ The means noted with same superscript (a, b, c, d, e, f) indicate there are no statistically significant difference among them, but there are statistically significant difference among means noted with different superscript, after correction for multiple comparisons using Turkey method.

Supplemental Figure 1.

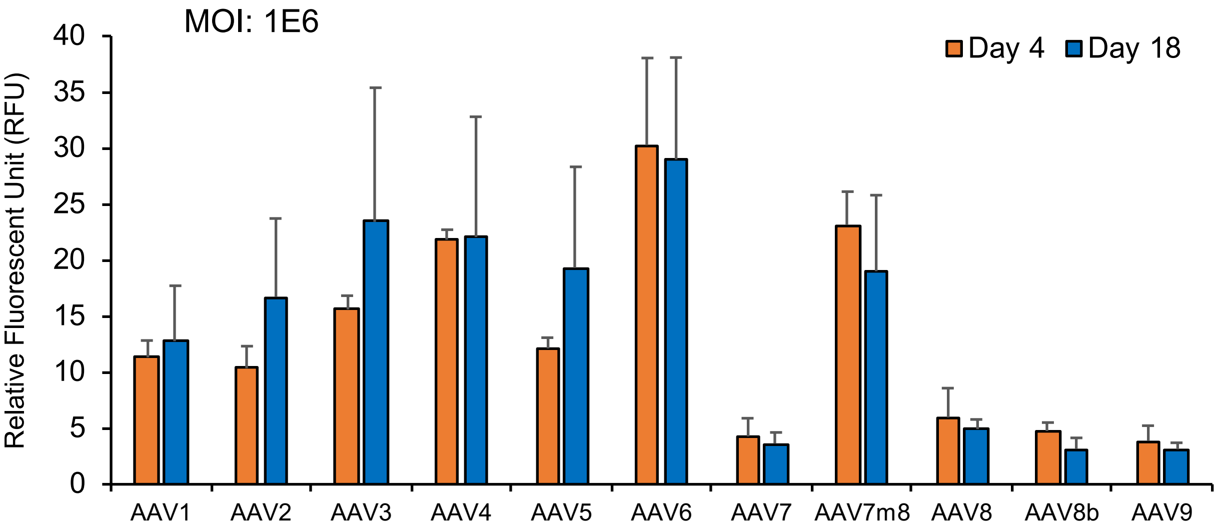

Supplemental Figure 2.

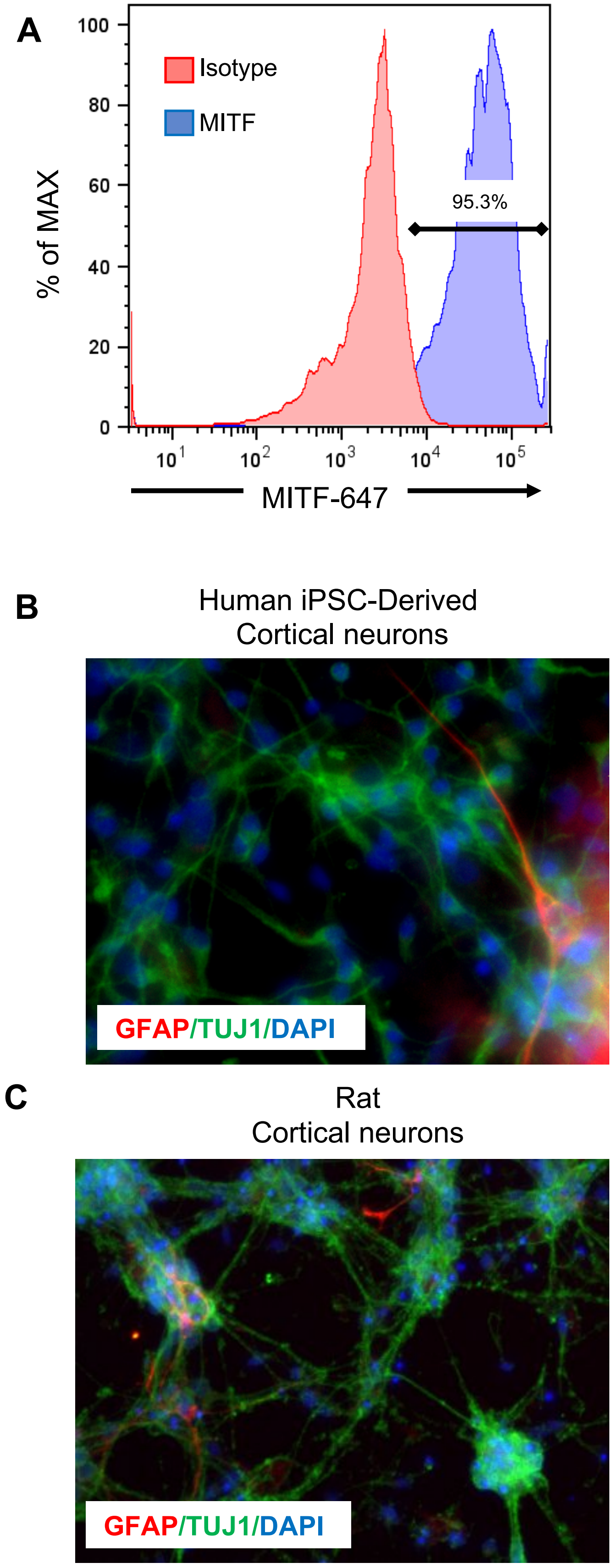

Supplement: Supplementary Materials — Supplemental Table 1: representative AAV dosage, titer, and MOI calculation for iPSC, RPE, and human and rat cortical cells. Supplemental Table 2: relative fluorescence intensity (R.F.U.) per cell at 96 h at MOI (1E6 vg/cell) Supplementary Figure 1: stable GFP transgene expression in RPE. (a) The AAVeGFP expression in RPE at d4 (96 h) and d18 for a dosage of 1E6 vg/cell shows similar expression in longer term cultures. There is no statistically significant difference between day 4 and day 18 in any of the AAV. Supplementary Figure 2: RPE and cortical neuron characterization. (a) Flow cytometric analysis of intracellular MITF protein expression. Greater than 95% of RPE expresses MITF. Plot showing MITF positive cells (blue) relative to mouse isotype control antibody (red). (b, c) Immunophenotyping of human and rat cortical neurons and astrocytes showing neuron-specific class III beta-tubulin (TUJ1; green) and glial fibrillary acidic protein (GFAP; red), respectively. Images were captured at 20x magnification (human iPSC cortical) and 10x magnification (rat cortical). [file 7281912.f1.pdf]
